# Differentiation of Mesenchymal Stem Cells Derived from Pancreatic Islets and Bone Marrow into Islet-Like Cell Phenotype

**DOI:** 10.1371/journal.pone.0028175

**Published:** 2011-12-16

**Authors:** Cristina Zanini, Stefania Bruno, Giorgia Mandili, Denisa Baci, Francesco Cerutti, Giovanna Cenacchi, Leo Izzi, Giovanni Camussi, Marco Forni

**Affiliations:** 1 Molecular Biotechnology Centre (MBC), University of Turin, Turin, Italy; 2 EuroClone S.p.A, Pero, Milan, Italy; 3 Research Centre for Experimental Medicine (CeRMS), University of Turin, Turin, Italy; 4 Department of Animal Production, Epidemiology and Ecology, University of Turin, Turin, Italy; 5 Clinical Department of Radiological and Histopathological Sciences, “Alma Mater Studiorum” University of Bologna, Bologna, Italy; 6 Department of Internal Medicine, University of Turin, Turin, Italy; University of Medicine and Dentistry of New Jersey, United States of America

## Abstract

**Background:**

Regarding regenerative medicine for diabetes, accessible sources of Mesenchymal Stem Cells (MSCs) for induction of insular beta cell differentiation may be as important as mastering the differentiation process itself.

**Methodology/Principal Findings:**

In the present work, stem cells from pancreatic islets (human islet-mesenchymal stem cells, HI-MSCs) and from human bone marrow (bone marrow mesenchymal stem cells, BM-MSCs) were cultured in custom-made serum-free medium, using suitable conditions in order to induce differentiation into Islet-like Cells (ILCs). HI-MSCs and BM-MSCs were positive for the MSC markers CD105, CD73, CD90, CD29. Following this induction, HI-MSC and BM-MSC formed evident islet-like structures in the culture flasks. To investigate functional modifications after induction to ILCs, ultrastructural analysis and immunofluorescence were performed. PDX1 (pancreatic duodenal homeobox gene-1), insulin, C peptide and Glut-2 were detected in HI-ILCs whereas BM-ILCs only expressed Glut-2 and insulin. Insulin was also detected in the culture medium following glucose stimulation, confirming an initial differentiation that resulted in glucose-sensitive endocrine secretion. In order to identify proteins that were modified following differentiation from basal MSC (HI-MSCs and BM-MSCs) to their HI-ILCs and BM-ILCs counterparts, proteomic analysis was performed. Three new proteins (APOA1, ATL2 and SODM) were present in both ILC types, while other detected proteins were verified to be unique to the single individual differentiated cells lines. Hierarchical analysis underscored the limited similarities between HI-MSCs and BM-MSCs after induction of differentiation, and the persistence of relevant differences related to cells of different origin.

**Conclusions/Significance:**

Proteomic analysis highlighted differences in the MSCs according to site of origin, reflecting spontaneous differentiation and commitment. A more detailed understanding of protein assets may provide insights required to master the differentiation process of HI-MSCs to functional beta cells based only upon culture conditioning. These findings may open new strategies for the clinical use of BM-MSCs in diabetes.

## Introduction

Type I diabetes is an immunologically-mediated disease with a genetic predisposition and results in the destruction of β-cells in pancreatic islets. Current therapy is based upon the long-life parenteral injection of insulin.

Although other therapeutic approaches such as pancreas or pancreatic islet transplantation may appear attractive, they are hampered by several difficulties (i.e. shortage of solid organs donors, immunosuppression, to avoid immunological rejection and long-lasting complications). Therefore transplantation is seldom used in clinical practice [1], [2].

Adult stem cells and their manipulation may open new perspectives for a radical therapeutic approach to type I diabetes [3].

Stem cell (SC) plasticity and their capability of being manipulated to induce differentiation, may allow the in vitro expansion of insulin-producing cells suitable for in vivo transplantation. Therefore, immune-mediated rejection could be avoided if insulin-secreting cells were obtained from the patient's own stem cells. A key issue for future clinical use of conditioned SC is represented by the site of origin that should be easily accessible and allow the harvesting of a stem cell population sufficient for in vitro manipulations and subsequent in vivo engrafting.

To date, several studies have reported experimental data on differentiation, from stem cells of varying origins, to islet-like cells (ILCs) [4].

Nevertheless, the process for induction of differentiation is not completely understood and may be influenced by different culture conditioning [3].

With the present study we report our experience on culturing mesenchymal stem cells (MSCs) derived from either pancreatic islets (HI-MSCs) or bone marrow aspirate (BM-MSCs), in a serum-free culture medium of our formulation, resulting in the production of insulin.

It has been demonstrated the presence of human islet-derived precursor cells that exhibit many characteristics of MSC [5] and that could be considered a source of beta-cells *ex vivo* production. Despite the presence of this resident MSC population in human islets, bone marrow may represent a potential source of MSC that is accessible for SC harvesting, as the hematological widespread transplantation practice demonstrate, especially if compared to pancreatic islets that are difficult to sample and, more relevant, seriously damaged or destroyed in diabetic patients.

We applied proteomic techniques to evaluate whether variations in protein expression in expanded and differentiated HI-MSCs and BM-MSCs are inherent to SC origin or whether they are influenced by the conditioning process.

Proteomic profiling of human pancreatic islet-cells has been reported, [6], [7] with the identification of 66 different proteins, serving as a reference map of human islet cell populations. These data were however at variance with the reported proteomic data on islet cells of murine and rat origin [8], [9].

A wealth of data, including proteomic studies, using cultured rat insulinoma cells were put forward, and were focused on selected insulin-secreting clones [10]. Sophisticated proteomic data on mouse and rat models of type II diabetes have been reported in the literature [11], [12].

In addition, a report of the proteomic profile of ovine BM-MSCs has been recently described, including comparisons with MSCs from other tissues of origin [13]. Nevertheless, detailed data comparing human MSCs of different origin have yet to be described, and no proteomic data have been reported on HI-MSCs. The comparison of the proteomic assets of HI and BM-MSCs may unravel similarities and differences correlated to the differentiation process, and it might provide new insights into the conditioning protocols required in order to achieve a stable and robust secretion of insulin.

## Results

### Characterization of BM- and HI-MSCs

BM-MSC and HI-MSC preparations satisfied the minimal criteria fixed by the International Society for Cellular Therapy to define a population as mesenchymal stromal cells [14], [15].

**Figure 1:**
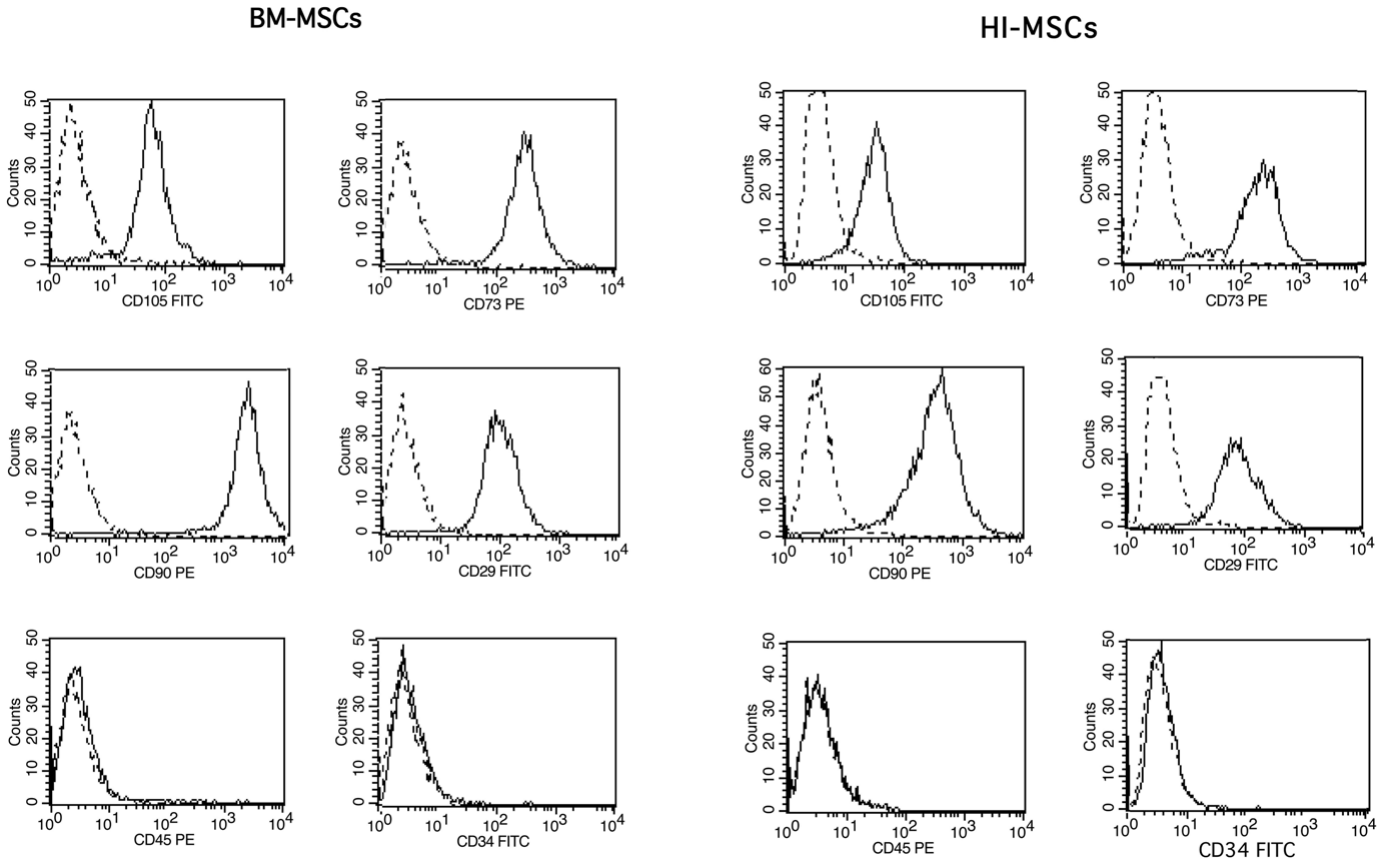
Characterization of BM and HI-MSCs. Representative FACS analyses shows that BM and HI-MSCs were positive for surface marker characterization of MSCs (CD105, CD73, CD90, CD29), and were negative for specific hematopoietic markers (CD45 and CD34) Dotted line is the isotypic control. All cell lines were tested with similar results.

Therefore, BM and HI-MSCs were plastic-adherent when maintained in expansion conditions; BM- and HI-MSC preparations expressed CD105, CD73, CD90 and CD29 (Figure 1 A and B), and they did not express hematopoietic markers, like CD45, CD14 and CD34.

**Figure 2:**
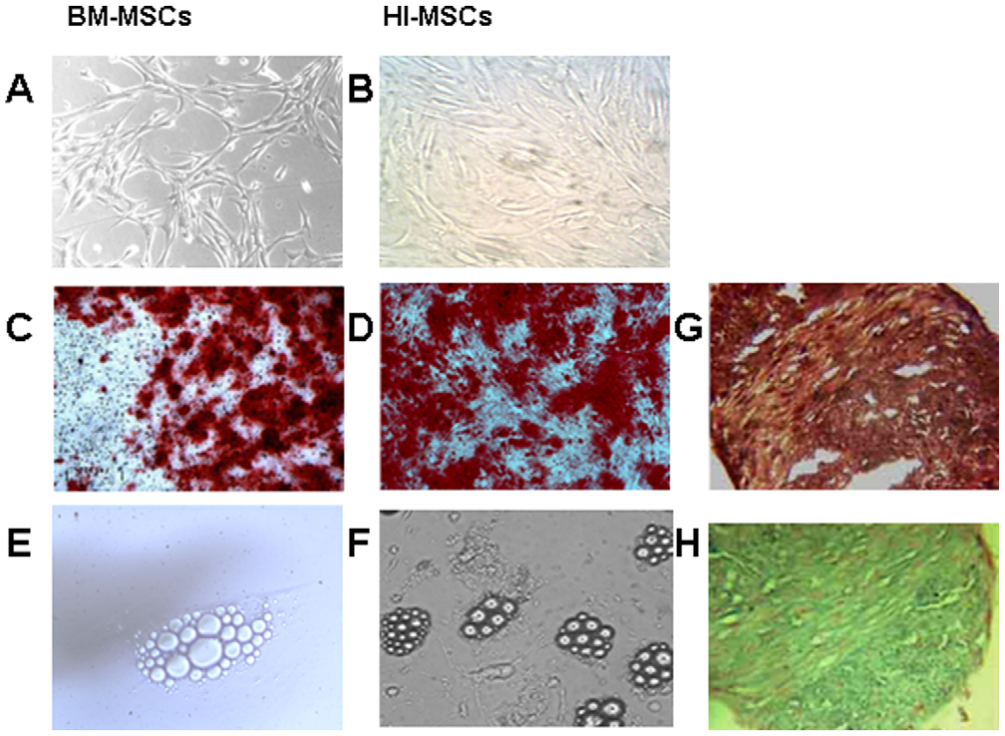
Multilineage differentiation of BM and HI-MSCs. (**A**) cultured BM-MSCs and (**B**) HI-MSCs before differentiation. (**C–D**) Representative micrographs of osteogenic differentiation: calcium depositions were detected by Alizarin Red after culturing BM-MSCs (**C**) and HI-MSCs (**D**) for 21 days in specific osteogenic medium (see Material and Methods). (**E–F**) Representative micrographs of adipogenic differentiation showing the presence of lipid droplets after 21 days in adipogenic differentiating medium in of BM-MSCs (**E**) and HI-MSCs (**F**) respectively. (magnification ×200). (**G and H**) Representative micrographs of chondrogenic differentiation shown by formation of a pellet positive for safranin O (**G**) and alcian blue (**H**) after culturing HI-MSC in chondrogenic medium for 28 days (magnification ×100). Five different lines were studied with similar results.

Concerning the ability to undergo differentiation into multiple mesenchymal lineages, BM-MSCs efficiently underwent osteogenic, adipogenic and chondrogenic differentiation, whereas HI-MSCs efficiently only underwent osteogenic and chondrogenic differentiation (Figure 2). Adipogenic differentiation of HI-MSCs was only observed in rare cells containing lipid droplets.

### Effect of culture media in stem cell morphology

**Figure 3:**
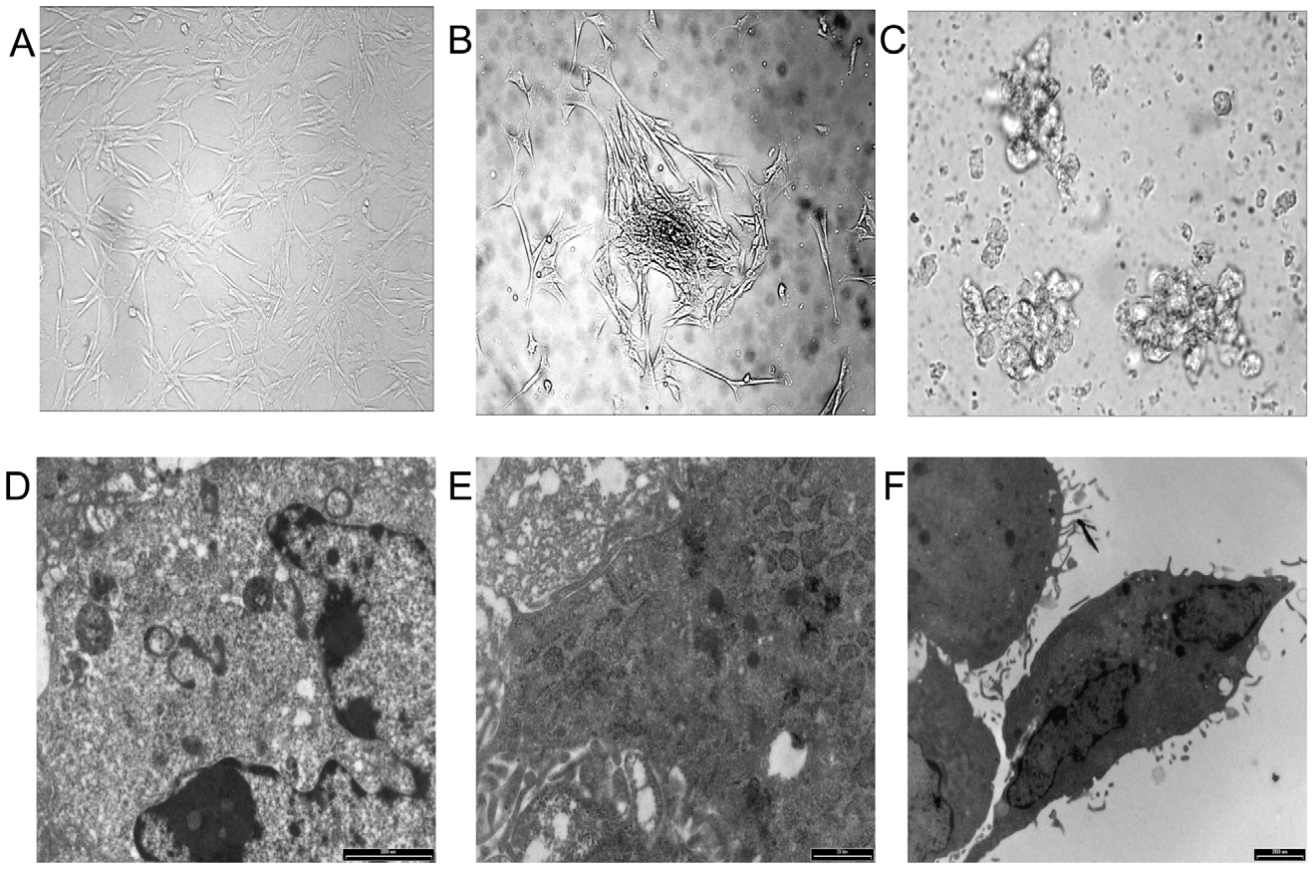
Morphological changes and ultrastructural analysis of BM and HI-MSCs during differentiation. (**A**) Representative HI-MSCs undifferentiated cells shows a fibroblast-like cell morphology (**B**) after 7 days in the complete differentiative medium HI-MSCs started to organize into small aggregates (**C**) finally, during maturation, HI-ILCs formed islet-like spatial structures. **Electron microscopy**: (**D**) HI-MSCs before differentiation showed stem cell features with undifferentiated cytoplasm and bean-shaped nucleus, (**E**) High magnification of a cytoplasm from a BM-ILCs derived cell showing numerous RER cisternae associated with some electron-dense core insulin-like granules. (**F**) HI- ILCs shows few secretory granules with a central electron-dense core and a peripheral halo resembling immature insulin-secreting granules.

**Figure 4:**
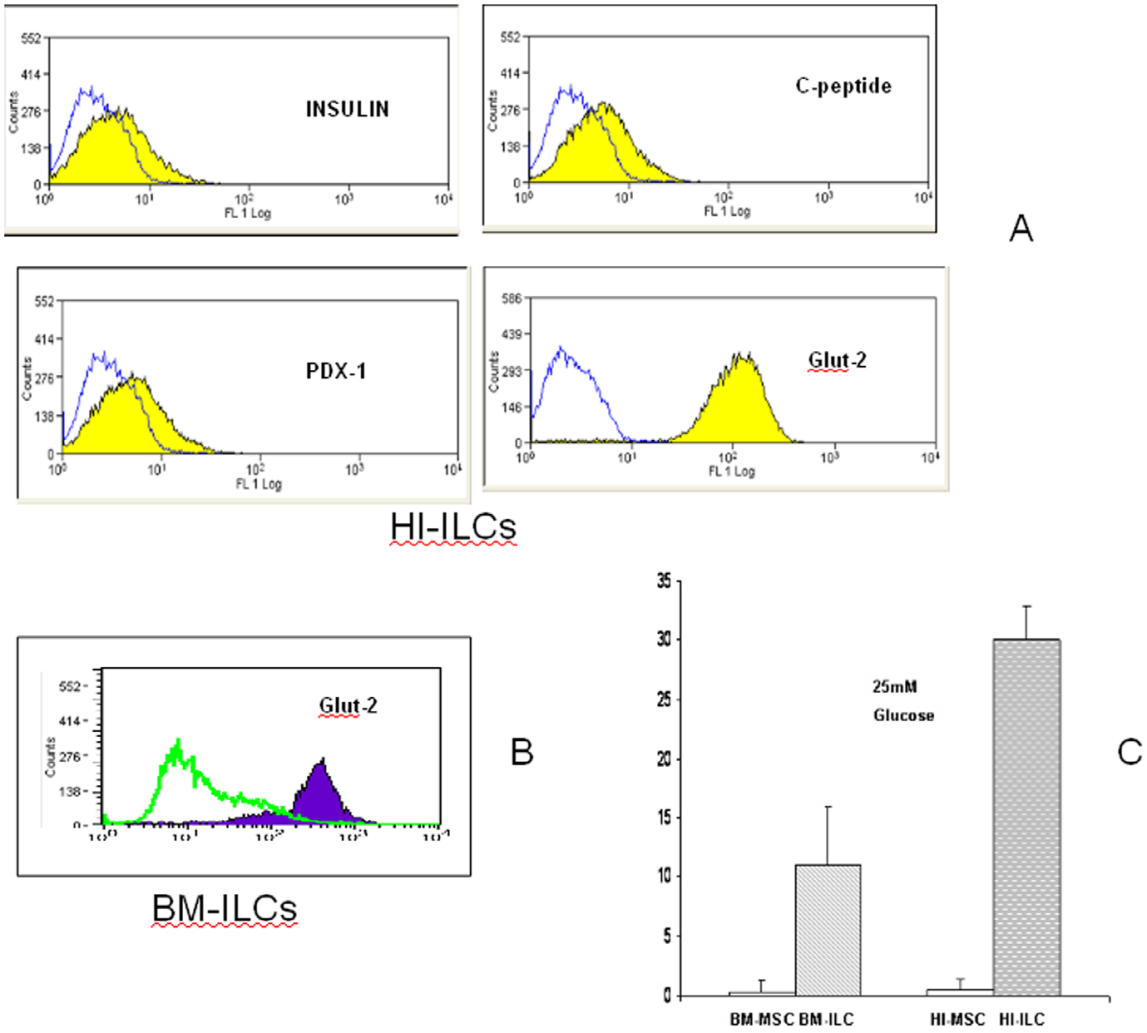
Analysis of Islet expression by Flow cytometry and measurement of insulin secretion by ELISA. (**A**) Representative FACS analyses shows that HI-MSCs were positive for markers characteristic of ILCs (Insulin, PDX-1, C-peptide and Glut-2) and that (**B**) BM-MSCs were only positive for Glut-2; (**C**) ELISA test shows insulin levels of supernatants from MSCs at basal level (BM-MSCs and HI-MSCs) and after differentiation to ILCs (BM-ILCs and HI-ILCs).

To investigate the effect on morphology by the differentiative medium, stem cells from different origins -HI-MSCs and BM-MSCs- were cultured with basal medium supplemented with platelet lysate (PL), retinoic acid, activin, GLPI-1, EGF, FGF, beta-cellulin, nicotinamide and glutamine. In this way differentiation of ILCs was induced for 3 weeks. In Figure 3 the undifferentiated MSCs assumed an adherent spindle, fibroblast-like cell morphology (A); they then differentiated to organize small aggregates (B) which led to maturation in islet-like cells (C). Ultrastructural analysis showed that undifferentiated MSCs are characterized by stem cells featuring a ribosome-rich undifferentiated cytoplasm and a bean-shaped nucleus (D); BM-MSC differentiated cells disclose numerous RER cisternae associated with some electron-dense core insulin-like granules (E); and finally, HI-MSC differentiated cells revealed a few secretory granules with a central electron-dense core and a peripheral halo resembling immature insulin-secreting granules (F). In Figure 4, cytofluorimetric analysis in HI-MSC differentiated cells showed the expression of ILC islet markers such as insulin, C-peptide, PDX-1 and Glut-2 (panel A); in BM-MSC differentiated cells only Glut-2 and insulin were expressed (panel B). Immunofluorescence on coverslips confirms that HI-ILC expressed insulin and PDX-1 (data not shown). ELISA tests on supernatants after 25 mM glucose stimulation (Figure 4 panel C) detected a robust secretion by HI-ILCs and an evident secretion by BM-ILCs. Both results are in the reference range for values from serum of healthy human donors (11–86 pmol/L – confidence interval 95% -). HI-ILCs showed almost a three-fold increase in insulin concentration in comparison to BM-ILCs (30 pmol for HI-ILCs versus 11 pmol for BM-ILCs). No detectable levels were found in HI-MSC and BM-MSC (insulin values below the detection limit of 3 pmol). Data suggest that the obtained ILCs represent a definitive step towards committed β-like cells sensitive to glucose challenge.

### Proteomics

**Figure 5:**
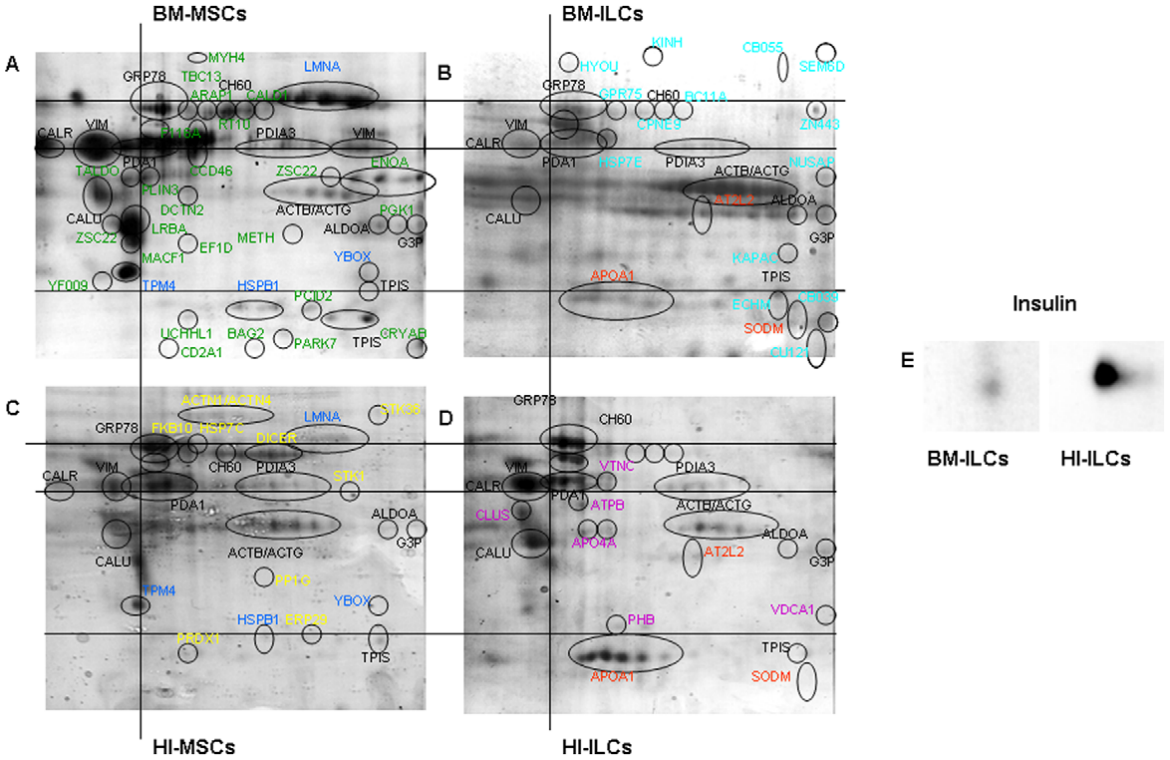
2-DE analysis of BM-MSCs and HI-MSCs treated or untreated with differentiative medium and 2-DE Western blot for Insulin. Representative image from three independent experiments of Coomassie blue stained 2-DE patterns of (**A**) BM-MSCs, (**B**) BM-ILCs, (**C**) HI-MSCs and (**D**) HI-ILCs. Proteins showing differential expression were indicated with different colours: in *black* -proteins identified in all cell lines-; in *blue*- proteins identified in basal cell lines; in *yellow*- proteins identified only in HI-MSCs; in *green-* proteins identified only in BM-MSCs; in *red-* proteins identified in ILCs obtained from HI-MSCs and BM-MSCs, in *violet* proteins identified only in HI-ILCs; in *light blue-* proteins identified only in BM-ILCs. Corresponding identifications are reported in Tables 1, 2, 3. In (**E**) Representative image of 2-DE Western blotting for Insulin detected in BM-ILCs and HI-ILCs.

Proteins from three independent biological replicas of MSCs and ILCs were extracted and analyzed by 2-DE using a nonlinear gradient of pH 3–10. Resulting gels, stained with Colloidal Coomassie blue G, were quantitatively and statistically analyzed by PDQuest as described above (see Material and Methods). Figure 5 shows representative 2-DE gel images, showing the differentially expressed spots identified in each gel, illustrated by different colors. Overall, we selected spots that were shared or unique in each cell line, and these were excised from gels and subjected to in-gel tryptic digestion and identification by MALDI-TOF, for a total of 315 spots. These proteins corresponded to 72 unique proteins, and in some case the same protein was identified in different spots over the gel (e.g. GRP78 or PDIA1 in Fig. 5). Among these 72 proteins, listed in Tables 1, 2, and 3, eleven proteins (indicated in black in Fig. 5 A–B–C–D) were shared by all cell lines. After statistical analysis of the normalized quantities of matched spots of 2-DE of these shared proteins, only six proteins (GRP78, PDA1, CALR, CALU, ACTB, ACTG) displayed significant differences (Table 1). Comparison between gels revealed evident differences in the qualitative and quantitative proteomic profile between the two populations of MSCs basal lines (panel A and C and Table 2). As shown in Figure 5 (panel C), there were 25 most expressed proteins in HI-MSCs; only four of these were shared with BM-MSCs (LMNA, TPM4, HSPB1, YBOX, indicated in blue), and ten were unique for these basal cells (indicated in yellow). Out of the forty proteins visualized in the BM-MSCs gel (panel A), twenty-four were unique for this basal cell population (indicated in green). This was more than twice the number found in HI-MSCs, probably due to a major undifferentiated state of the BM-MSCs compared to a partial committed state of basal HI-MSCs. After the ILCs differentiation (panel B and D and Table 3) with the identical conditioning process, a different *repertoire* of proteins was generated. Both types of ILCs were characterized by three new proteins, APOA1, ATL2 and SODM (indicated in red), while some remained unique to the individual differentiated cells lines. APOA1 was more greatly expressed in HI-ILCs compared to BM-ILCs, a difference that was statistically significant. In particular, new proteins were expressed: fourteen proteins (indicated in light blue) unique to BM-ILCs, and six unique to HI-ILCs (indicated in violet). 2-DE Western blotting for Insulin detected the hormone in both cell populations, confirming the immunological data. Figure 5 (panel E) shows that insulin expression was higher in HI-MSC differentiated cells compared to the BM counterpart.

**Table 1:** Proteins shared by MSCs and ILCs, as identified by MALDI-TOF MS.

Proteins identified in all cell populations (HI-MSCs; BM-MSCs, HI-ILCs, BM-ILCs)									
Acc.Numb	Ab.Name	Full Name	MW(Da)^a^	pI^b^	MP^c^	C%^d^	MS^e^	Ratio^f^	*p*
O43852	CALU	Calumenin	37198	4,47	7	28	70	1,1±0,5	S*
P27797	CALR	Calreticulin	48283	4,29	10	32	114	1,5±0,3	S*
P10809	CH60	60 kDa heat shock protein, mitochondrial	61187	5,7	9	21	80	1,7±0,6	NS
P11021	GPR78	78 kDa glucose-regulated protein	72402	5,07	16	35	185	3,7±0,1	HS**
P07237	PDIA1	Protein disulfide-isomerase	57480	4,76	10	27	111	2,5±0,3	S*
P30101	PDIA3	Protein disulfide-isomerase A3	57146	5,98	11	29	124	2,1±1,7	NS
Q96HG5	ACTB	Actin, cytoplasmic 1	42052	5,29	13	38	149	3,1±0,2	S*
P63261	ACTG	Actin, cytoplasmic 2	42108	5,31	13	38	149	3,1±0,2	S*
P04406	G3P	Glyceraldehyde-3-phosphate dehydrogenase	36201	8,57	6	27	63	1,1±1,6	NS
P60174	TIPS	Triosephosphate isomerase	26938	6,45	15	67	233	0,7±0,5	NS
Q96ML2	VIM	Vimentin	53676	5.06	19	45	210	2,1±1,1	NS

a MW, molecular weight.

b pI, isoelectric point.

c Number of matched mass values on number of total mass values searched.

d The sequence coverage, which is calculated as the percentage of identified sequence to the complete sequence of the matched protein.

e Mascot Score.

f Ratio between level of spot avarage expression indicated in Fig. 5 Standard deviation is indicated. For the significance two-sided Student's *t* test was used (*p<0.05,**p<0.01).

**Table 2:** Proteins differentially expressed in MSCs as identified by MALDI-TOF MS.

Proteins identified in basal cell populations (HI-MSCs;BM-MSCs)									
Acc.Numb	Ab.Name	Full Name	MW(Da)^a^	pI^b^	MP^c^	C%^d^	MS^e^	Ratio^f^	*p*
Q9UC36	HSPB1	Heat shock protein beta-1	22826	5,98	8	42	121	1,3±0,9	NS
P02545	LMNA	Prelamin-A/C	74380	6,57	17	27	170	2,1±1,3	NS
Q9UCS3	TPM4	Tropomyosin alpha-4 chain	28619	4,67	8	31	91	1,5±0,9	NS
P67809	YBOX	Nuclease-sensitive element-binding protein 1	35903	9,87	8	19	65	0,9±0,8	NS

Proteins identified only in HI-MSCs									
Acc.Numb	Ab.Name	Full Name	MW(Da)^a^	pI^b^	MP^c^	C%^d^	MS^e^	Ratio^f^	*p*
P12814	ACTN1	Alpha-actinin-1	103563	5,25	10	13	72	_	_
O43707	ACTN4	Alpha-actinin-4	105245	5,27	12	16	98	_	_
Q9UPY3	DICER	Endoribonuclease Dicer	221279	5.47	12	20	56	_	_
P30040	ER29	Endoplasmic reticulum resident protein 29	29032	6,77	6	28	67	_	_
Q96AY3	FKB10	Peptidyl-prolyl cis-trans isomerase FKBP10	64717	5,36	8	17	78	_	_
P11142	HSP7C	Heat shock cognate 71 kDa protein	71082	5,37	8	17	103	_	_
Q13162	PRDX4	Peroxiredoxin-4	30749	5,86	8	43	94	_	_
P36873	PP1G	Serine/threonine-protein phosphatase PP1-gamma catalytic subunit	37701	6,12	7	27	72	_	_
Q9NRP7	STK36	Serine/threonine-protein kinase 36	143995	5.57	8	20	53	_	_
Q6J9G0	STYK1	Tyrosine-protein kinase STYK1	47576	7.10	8	15	40	_	_

Proteins identified only in BM-MSCs									
Acc.Num	Ab.Name	Full Name	MW(Da)^a^	pI^b^	MP^c^	C%^d^	MS^e^	Ratio^f^	*p*
Q96L71	ARAP1	Arf-GAP with Rho-GAP domain,	163743	5,86	9	10	68	_	_
O95816	BAG-2	BAG family molecular chaperone regulator 2	23928	6,25	7	32	72	_	_
Q05682	CALD1	Caldesmon	93251	5,63	8	12	57	_	_
Q8N8E3	CCD46	Coiled-coil domain-containing protein 46	113192	6,25	9	11	56	_	_
P42771	CD2A1	Cyclin-dependent kinase inhibitor 2A, isoforms 1/2/3	16579	5,52	4	28	60	_	_
P02511	CRYAB	Alpha-crystallin B chain	20146	6,76	9	45	140	_	_
P29692	EF1D	Elongation factor 1-delta	31217	4,9	9	37	104	_	_
Q6GMP2	ENOA	Alpha-enolase	47481	7,01	11	39	151	_	_
Q8IWF6	FF116A	Protein FAM116A	70215	8,52	6	13	59	_	_
P50851	LRBA	Lipopolysaccharide-responsive and beige-like anchor protein	321586	5,39	14	7	68	_	_
Q9UPN3	MACF1	Microtubule-actin cross-linking factor 1, isoforms 1/2/3/5	623626	5,27	16	3	56	_	_
Q99707	METH	Methionine synthase	141749	5,39	9	9	56	_	_
Q9Y623	MYH4	Myosin-4	223902	5,65	11	7	56	_	_
Q99497	PARK7	Protein DJ-1	20050	6,33	7	28	70	_	_
Q9NVY1	PCID2	PCI domain-containing protein 2	46627	8,78	5	12	56	_	_
P00558	PGK1	Phosphoglycerate kinase 1	44985	8,3	7	18	70	_	_
P37837	TALDO	Transaldolase	37688	6,36	6	24	57	_	_
Q9NVG8	TBC13	TBC1 domain family member 13	47151	5,11	5	19	59	_	_
P09936	UCHL1	Ubiquitin carboxyl-terminal hydrolase isozyme L1	25151	5,33	6	31	67	_	_
Q6ZS46	YF009	Putative uncharacterized protein FLJ45840	22478	10,58	5	34	63	_	_
P82664	RT10	28S ribosomal protein S10, mitochondrial	23099	7,78	6	32	63	_	_
O60664	PLIN3	Perilipin-3	47189	5,3	7	23	82	_	_
Q13561	DCTN2	Dynactin subunit 2	44318	5,1	6	18	52	_	_
P10073	ZSC22	Zinc finger and SCAN domain-containing protein 22	55724	7,36	6	18	64	_	_

a MW, molecular weight.

b pI, isoelectric point.

c Number of matched mass values on number of total mass values searched.

d The sequence coverage, which is calculated as the percentage of identified sequence to the complete sequence of the matched protein.

e Mascot Score.

f Ratio between level of spot avarage expression indicated in Fig. 5 Standard deviation is indicated. For the significance two-sided Student's *t* test was used (*p<0.05,**p<0.01).

**Table 3:** Proteins differentially expressed in ILCs as identified by MALDI-TOF MS.

Proteins identified in ILCs obtained from HI-MSCs and BM-MSCs with conditioned media									
Acc.Numb	Ab.Name	Full Name	MW(Da)^a^	pI^b^	MP^c^	C%^d^	MS^e^	Ratio^f^	*p*
P02647	APOA1	Apolipoprotein A-I	30759	5,56	12	37	147	3,2±0,1	HS**
P04179	SODM	Superoxide dismutase [Mn], mitochondrial	24878	8,35	6	30	72	1,1±0,8	NS
Q8IUZ5	AT2L2	Alanine–glyoxylate aminotransferase 2-like	50135	6,28	7	18	68	2,1±0,9	NS

Proteins identified only in HI-ILCs									
Acc.Num	Ab.Name	Full Name	MW(Da)^a^	pI^b^	MP^c^	C%^d^	MS^e^	Ratio^f^	*p*
P04004	VTNC	Vitronectin	55069	5,55	8	16	76	_	_
P21796	VDCA	Voltage-dependent anion-selective channel protein 1	30868	8,62	6	31	110	_	_
P06727	APO4A	Apolipoprotein A-IV	45371	5,28	12	29	21	_	_
P10909	CLUS	Clusterin	53031	5,89	7	22	76	_	_
P35232	PHB	Prohibitin	29843	5,57	6	27–34	102	_	_
P06576	ATPB	ATP synthase subunit beta, mitochondrial	56525	5,26	10	29	109	_	_

Proteins identified only in BM-ILCs									
Acc.Num	Ab.Name	Full Name	MW(Da)^a^	pI^b^	MP^c^	C%^d^	MS^e^	Ratio^f^	*p*
Q96MZ4	CB039	Uncharacterized protein C4orf39	17295	11,82	5	53	64	_	_
P30084	ECHM	Enoyl-CoA hydratase, mitochondrial	31823	8,34	5	25	59	_	_
Q9UC56	GPR75	Stress-70 protein, mitochondrial	73920	5,87	8	16	68	_	_
Q0VDF9	HSP7E	Heat shock 70 kDa protein 14	55444	5,41	7	19	65	_	_
P17612	KAPAC	cAMP-dependent protein kinase catalytic subunit alpha	40678	8,84	6	19	58	_	_
P33176	KINH	Kinesin-1 heavy chain	110358	6,12	8	11	56	_	_
Q9BXS6	NUSAP	Nucleolar and spindle-associated protein 1	49593	9,92	5	16–28	60	_	_
Q9H165	BC11A	B-cell lymphoma/leukemia 11A	92565	6,15	8	11	56	_	_
Q6NV74	CB055	Uncharacterized protein C2orf55	102665	8,13	6	10	56	_	_
Q8IYJ1	CPNE9	Copine-9	62281	5,18	7	16	59	_	_
Q8N0V1	Cu121	Putative uncharacterized protein C21orf121	14961	9.08	3	31	47	_	_
Q9Y2A4	ZN443	Zinc finger protein 443	80145	9.30	7	12	60	_	_
Q9P249	SEM6D	Semaphorin-6D	121050	8,77	8	9	56	_	_
Q9Y4L1	HYOU1	Hypoxia up-regulated protein 1	111494	5,16	7	8	56	_	_

a MW, molecular weight.

b pI, isoelectric point.

c Number of matched mass values on number of total mass values searched.

d The sequence coverage, which is calculated as the percentage of identified sequence to the complete sequence of the matched protein.

e Mascot Score.

f Ratio between level of spot avarage expression indicated in Fig. 5 Standard deviation is indicated. For the significance two-sided Student's *t* test was used (*p<0.05,**p>0.01).

The comparison of our data with the proteomic reference map of human islet cells [6] is only tentative as different cell lines are present in pancreatic islets.

Nevertheless, several of the proteins identified in HI-ILCs are in accordance with the islet reported data (GAPD, GRP78, HSP 60 ATP beta chain, PDI, Calreticulin and ACTB), while others present in the basal MSCs (like Elongation factor 1, HSP 70 and α-enolase) are lost following induction of differentiation. Other proteins are expressed by conditioned cells and are not found in islet cells. However, a more stringent comparison is hampered by the fact that islet cells from the human pancreas are a composite population of different endocrine cells and not only beta-cells.

### Hierarchical Cluster analysis

**Figure 6:**
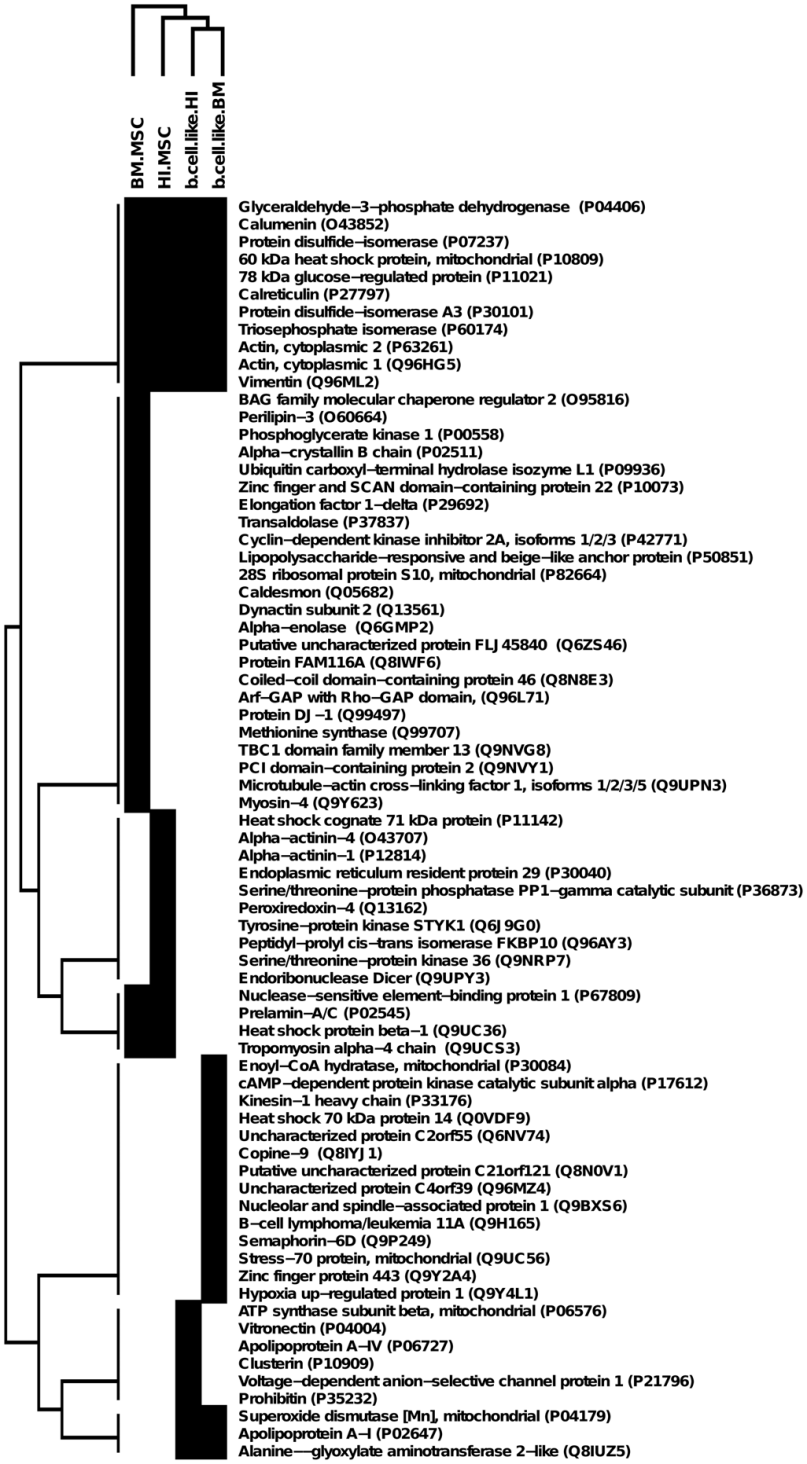
Hierarchical Cluster Analysis. This figure shows a cluster map of the proteins expressed in the different cell lines. The tree on the left represents the hierarchical cluster analysis on the 72 proteins. In the heat map, black squares show the expression of the corresponding protein in the correspondent cell lines, while white squares show no expression.

The expression profiling of the identified proteins (reported in the heat map in Fig. 6), underlines the groups of proteins uniquely expressed by the four cell types, as well as those expressed by both stem cell lines or both differentiated cell lines, or all the different cell types. The hierarchical cluster also reveals a marked difference between the proteins expressed by the basal cell lines and the differentiated ones. As indicated by the tree (Figure 6), the two cell lines lie on two separated branches, demonstrating that their expression profiles are dissimilar.

## Discussion

In the effort of making SC of different origins suitable for clinical use in human patients, the key role of four genes (OCT4 KLF4 SOX2 and c-Myc) emerged as an effective approach to increasing pluripotency through reprogramming [16], [17]. A recent report described the induction of MSCs from renal mesangial cells by transfection for these factors [18].

While the reprogramming of MSCs is emerging as a pivotal element, several divergent strategies were developed to limit or avoid harsh manipulations that, although effective, may be prone to side effects, hampering the safety of such interventions.

An innovative approach was utilized by a recent report [19]. The described results rely upon the recognition of the constitutional activation of one or more of the above factors in skin-derived SC from the dermal papilla of hair follicles.

Several different strategies and innovative ways were put forward to induce less drastic cellular modifications, based on limited genetic manipulation and controlled expression of transforming factors, [20] such as the use of micro RNAs, and small molecules acting as stimulators or inhibitors of definite signaling pathways that may open new effective approaches [21].

The inductive role of the microenviroment may also be important in order to achieve maturation to functional beta cells, and may possibly be fully exploited in vivo. Interestingly, in the recent report by Karniel, BM-MSC transfected with rat PDX1 produced insulin in vitro, and showed transcription of human PDX1 when transplanted in kidney capsules of immunodeficient mice [22].

Conditioning by means of a differentiation medium has several limitations. Nevertheless, a more detailed description of protein modifications induced only by culture media conditioning may constitute the basis for a rational and safe manipulation of stem cells derived from different sources.

The search for small molecules that interfere with signalling pathways may open new useful opportunities for clinical usage in several human disease conditions.

Our study was aimed to clarify modification of MSCs of different origin towards pancreatic beta cell differentiation via culture media induction of differentiation.

Modification of the protein asset during these medium-induced modifications may be of interest in order to correlate the regulatory mechanisms acting in the differentiation process. Moreover, it may possibly give an insight into the medium-induced SCs modifications that would be useful for a better control of cell manipulations through safe and conservative approaches.

Proteomic analysis enhanced the differences in the MSCs related to the site of origin, reflecting spontaneous differentiation and commitment.

The conditioning media produced modifications of protein expression in both basal MSCs, with partially similar results, and a reduction in the number of proteins expressed. It is not surprising that 2-DE analysis failed to detect Insulin, probably due to its low molecular weight (5808 Da) which has also been reported in the literature [9]. Insulin was however, detectable in 2-DE Western blotting from cell populations after differentiation, and in supernatants of ILCs after glucose stimulation, as demonstrated by ELISA analysis.

A limited number of proteins were present in ILCs with β phenotype, and the original controls were PDIA1, PDIA3, GPR78, vimentin, CH60 and triphosphates isomerase. The differentiated cells maintained a proteomic profile restricted to the cell type of origin, and expressed a specific set of new proteins. Three new proteins, APOA1, ATL2 and SODM (indicated in red), were present in both ILC types.

APO A1 is a protein involved in lipid transport and metabolism; it is also a constituent of the cell membrane, and it may have a critical role in beta cells in the regulation of lipid metabolism in tapering fatty acid toxicity towards islet beta-cells [23]. In addition it has been related to chronic glucose stimulation, as seen in an insulinoma cell model, and in diabetes type II [24].

The role of ATL 2 may be relevant as it belongs to a class of proteins mediators of homotypic fusion of endoplasmic reticulum membranes [25], and the absence of ATL 1 is involved in the pathogenesis of hereditary spastic paralysis [26].

SODM (mitochondrial superoxide dismutase) is an essential component of the cellular defense mechanism against oxidative stress (ROS), which contributes to the damage to beta cells that plays a role in the pathogenesis of type 2 diabetes [27].

Besides the morphological similarity with the formation of an “islet-like” growth pattern induced by conditioning media, relevant differences of the proteomic asset were still present and, more importantly, while HI-MSCs were induced to secrete insulin and to produce evident secretory granules, conditioned cells derived from BM-MSCs produced only small quantities of insulin and, although formed islet-like agglomerates, maintained a more undifferentiated phenotype, as also determined by transmission electron microscopy.

The conditioning approach, so far, is effective in inducing insulin production by HI-MSCs, and further improvements may be forecast in this direction [22]. As enhanced by hierarchical cluster analysis and well-depicted in the heat map, culture conditioning was able to modify the protein asset of both basal cell lines, but towards apparently divergent differentiation pathways. This phenomenon may underscore the actual limits of a culture conditioning approach that may stimulate some degree of differentiation, but not change the direction of this process. However rational manipulation of culture conditions and the quest for possible inducing molecules might open new perspectives for the use of MSC from easily accessible sources for clinical usage in human pathology.

## Materials and Methods

### Chemicals and reagents

Liver Digest Medium was purchased from Gibco. Minimum essential medium/endothelial cell basal medium-1 (α-MEM/EBM), Alizarin Red, Transforming Growth Factor β3 were purchased from Lonza (Basel, Switzerland). Streptomycin, penicillin, protease inhibitors, benzonase, ammonium persulfate (APS), bromophenol blue, glycerol, N,N,N′,N′-tetramethylethylene-diamine (TEMED), sodium dodecyl sulphate (SDS), TRIZMA, urea, 3-[(3-cholamidopropyl) dimethylammonio]-1-propanesulphonate (CHAPS), dithiothreitol (DTT), iodoacetamide, Oil Red O, safranin O, alcian blue, nicotinammide, were purchased from Sigma-Aldrich (St. Louis, MO, USA). Activin, Glucagon-like peptide I (GLPI-1), Epidermal growth factor (EGF), Fibroblast growth factor (FGF), Beta-cellulin were purchased from PeproTech Inc.( Rocky Hill, NJ) DC Protein assay kit, acrylamide, agarose, ready-made immobilized pH gradient (IPG) strip (7-cm IPG strips pH 3–10NL and pH 4–7), were purchased from Bio-Rad (Hercules, CA, USA). Ampholine pH 3.5–10 and 5–8 were obtained from GE Healthcare (MI, ITALY). Fetal bovine serum, DMEM-low glucose, glutamine, Adipogenic, Osteogenic and Chondrogenic differentiation kits, EuroMed Human Mesenchymal Stem Cell Kit, nitrocellulose membrane, enhanced chemiluminescence kit were purchased from EuroClone (MI, ITALY). All phycoerythrin (PE) or fluorescein isothiocyanate (FITC) conjugated anti-CD105, -CD29, CD14, -CD31, -CD146, -CD44, -CD90 (Dakocytomation, Copenhagen, Denmark); -CD73, -CD34, -CD45, -CD80, -CD86,-CD166, HLA-I (Becton Dickinson Biosciences Pharmingen, San Jose, CA); -CD133 (Miltenyi Biotec, Auburn); KDR (R&D Systems, Abington, U.K.); -HLA-II (Chemicon International Temecula, CA), -CD40 (Immunotech, Beckman Coulter), -CD154 (Serotec, Raleigh, NC USA); Insulin, PDX1, GLUT-2, and secondary antibodies Anti-Rabbit IgG Alexa Fluor 488 conjugate, Anti-Mouse IgG Alexa Fluor 488 conjugate were purchased from Cell Signalling Technology, Inc. (Danver, MA).

### Isolation and characterization of human Bone Marrow and Islet-derived Mesenchymal Stromal Cells (BM-MSCs and HI-MSCs)

Approval of the study was obtained from the Center for Molecular Biotechnology Institutional Review Board. BM-MSCs were obtained from Lonza (Basel, Switzerland), cultured and characterized as previously described [15]. In brief, to expand the bone marrow cells, the adherent monolayer was detached by trypsin treatment for 5 minutes at 37°C, after 15 days for the first phase and every 7 days for each successive step. Cells were seeded at a density of 10,000 cells/cm^2^ and used during passage six. At each passage, cells were counted and analyzed for immunophenotype by cytofluorimetric analysis.

HI-MSCs, human pancreatic islets were obtained from the laboratory of islet transplantation (San Giovanni Battista Molinette Hospital, Torino). All tissue donors gave written, informed consent (according to procedures approved by Ethical Committee of San Giovanni Battista Hospital) for use of tissues for scientific research. Islets were purified according to Ricordi's protocol [2] from pancreases discarded for transplantation, and digested by treatment for 15 minutes at 37°C with Liver Digest Medium. The obtained suspension was then collected at the bottom of a conical tube by spontaneous precipitation, and the digested islets were transferred to fibronectin-coated T25 flasks in the presence of Minimum essential medium/endothelial cell basal medium-1 (α-MEM/EBM) (3∶1) supplemented with penicillin (50 IU/ml), streptomycin (50 mg/ml) and 10% of Foetal Calf Serum. Within 4 days, an islet outgrowth of adherent, monomorphic and spindle-shaped cells was observed. Confluence was achieved by day 7–8 when the cell monolayer was detached by trypsin–EDTA treatment. The remnants of the islets were removed by low-speed centrifugation and cells were expanded in the same medium at a concentration of 10,000 cells/cm^2^. At each passage, cells were counted and analyzed for immunophenotype by cytofluorimetric analysis.

The adipogenic, osteogenic and chondrogenic differentiation ability of BM and HI-MSCs was determined as previously described [14]. Briefly, BM-MSC and HI-MSCs were cultured with an Adipogenic differentiation kit for 3 weeks. To evaluate differentiation, cells were fixed with 4% paraformaldehyde for 20 minutes at room temperature, and stained with 0.5% Oil Red O in methanol for 20 minutes at room temperature.

Osteogenic differentiation was assessed by culturing BM-MSCs and HI-MSCs using an Osteogenic differentiation kit. Medium was changed 2 times a week, for 3 weeks. To evaluate differentiation, cells were fixed with 4% paraformaldehyde for 20 minutes and stained with Alizarin Red, pH 4.1 for 20 minutes at room temperature.

### MSCs differentiation

For chondrogenic differentiation, 2.5×10^5^ HI-MSCs and BM-MSCs were centrifuged in a 15-ml conical polypropylene tube (Falcon BD Bioscience) at 150 g for 5 minutes and washed twice with DMEM. BM-MSCs and HI-MSCs pellets were cultured using a Chondrogenic differentiation kit supplemented with 10 ng/ml of Transforming Growth Factor β3. Medium was changed every 3 days for 28 days. Pellets were fixed in 4% paraformaldehyde overnight, and paraffin-embedded sections were stained for glycosaminoglycans using 0.1% safranin O and for sulfated proteoglycans with 1% alcian blue.

### Flow Cytometry

The following antibodies, all phycoerythrin (PE) or fluorescein isothiocyanate (FITC) conjugated, were used for cytofluorimetric analyses: anti-CD105, -CD29, CD14, -CD31, -CD146, -CD44, -CD90, -CD73, -CD34, -CD45, -CD80, -CD86,-CD166, HLA-I, -CD133; KDR, -HLA-II, -CD40, -CD154 monoclonal antibodies. Mouse IgG isotypic controls were from Dakocytomation.

The following panel of Alexia Fluor conjugated antibodies was used: Insulin, PDX1, GLUT-2. Conjugated antibodies were incubated for 4 minutes in phosphate buffer saline (PBS) with 0.1% of Triton X 100. For each determination at least 10,000 cells were analyzed on a FACSCALIBUR cytometer (BD). Cell Quest (BD) software was used for result evaluation, including dot-plot and percentage of positive cells.

### Procedure to differentiate the MSCs populations into ILCs

HI-MSCs and BM-MSCs were cultured by seeding 1×10^5^ cells in T75 flasks using a EuroMed Human Mesenchymal Stem Cell Kit and grown at 80% of confluence, then differentiated towards β-cells like cells with the following medium: DMEM-low glucose, Platelet Lysate (PL) 5%, retinoic acid 10 µM (for 24 hours only), Activin 10 µg/ml, Glucagon-like peptide I 200 µg/ml (GLPI-1), Epidermal growth factor 20 ng/ml (EGF), Fibroblast growth factor 10 ng/ml (FGF), Beta-cellulin 10 µg/ml, nicotinammide 10 mM/L, glutamine 2 mM.

Cultures were using adherent conditions for the first 7 days then changed to ultra low attachment dishes (Corning) for a further 2 weeks.

### Immunofluorescence

Indirect immunofluorescence was performed on differentiated cells after 2% paraformaldehyde fixation. Cells were permeabilized with methanol (MetOH) for some antigens.

Primary antibodies were diluted according to manufacturer's instructions. The following antisera were utilized: pancreatic duodenal homeobox gene-1 (PDX1), Insulin, C Peptide. Secondary antibodies were Anti-Rabbit IgG Alexa Fluor 488 conjugate, Anti-Mouse IgG Alexa Fluor 488 conjugate. Non-immune rabbit or mouse sera were used as negative controls at a 1∶50 dilution.

### Ultrastructural analysis

HI-MSCs and BM-MSCs were fixed in 2.5% sodium cacodylate-buffered glutaraldehyde immediately after in vitro differentiation. Pelleted cells were post-fixed in osmium tetroxide in the same buffer, dehydrated in ethanol and embedded in Araldite. Thin sections stained with uranyl acetate and lead citrate were studied in a Philips 400T transmission electron microscope.

### Insulin secretion by ELISA

Supernatants from MSCs (HI-MSCs and BM-MSCs) and ILCs (HI-ILCs and BM-ILCs) at standard culture conditions (i.e. prior to glucose challenge) and after stimulation with 25 mmol/l glucose for 2 hours were collected and frozen at −80°C. A non-conditioned medium was used as a negative control for secreted insulin measurement. The insulin release was detected by Insulin ELISA kit (Dako, Italy) according to the manufacturer's instructions.

### Sample preparation for proteomics

HI-MSCs and BM-MSCs cell lines, control and differentiated cells, were collected directly from flasks and washed twice in PBS. Cells lysates were obtained using 2DE lysis solution (8 M urea, 4% w/v CHAPS, 40 mM Tris) with added protease inhibitor and benzonase (Sigma) by gentle lysis methods (freeze-thaw lysis) and shaking for 24 hours at room temperature.

Suspensions were centrifuged at 12,000 rpm for 15 min at 4°C, and the supernatants were conserved for 2-DE analysis. Protein concentrations were measured using a DC Protein Assay Kit.

### 2-DE electrophoresis analysis

The 7 cm ready-made IPG strips, pH 3–10 NL, were rehydrated in the passive mode with a 125 µl total volume contains 150 µg of protein, 2.5% (v/v) ampholine (3–10) and 1%(w/v) DTT. Western blotting for Insulin was performed with 300 ug of protein in the 7 cm ready-made IPG strips, pH 4–7. Isoelectric focusing was carried out in a Protean IEF cell apparatus (Bio-Rad).

In brief, focusing for the 7 cm IPG strips was started at 250 V; voltage was progressively increased up to 4000 V until a maximum of 25000 V-h was reached. Focusing was performed at 18°C with a limit of 50 µA per strip. Once the first dimension separation was terminated, the strip was equilibrated in two steps. The first with 50 mM Tris-HCl, pH 8.8, Urea 6 M, Glycerol 30% (v/v), SDS 2% (w/v) and DTT 1% (w/v) for 15 minutes, and the second step with the same buffer and the same time but with 2.5% (w/v) iodoacetamide instead of DTT.

For the second dimension separation, 10% vertical SDS-polyacrylamide gels were used.

Gels were stained with colloidal Coomassie Blue (18% v/v ethanol, 15% w/v ammonium sulphate, 2% v/v phosphoric acid, 0.2% w/v Coomassie G-250) for 48 hours, destained with water, and scanned using PDQuest software (version 7.2, Bio-Rad); all spots were processed for matrix-assisted laser desorption/ionization mass spectrometry (MALDI-TOF) analysis.

### 2-DE Western blotting

The small format gel was transferred to a nitrocellulose membrane. Membranes were blocked with 3% w/v BSA in PBS/Tween 20 0.1% (v/v) for 1 hour; membranes were then washed three times with PBS and probed with a 1∶200 dilution of anti-Insulin antibody O.N. at 4°C. Membranes were washed six times with PBS, incubated for 1 hour with a 1∶1000 dilution of anti-rabbit IgG peroxidase-labeled antibody and immuno-reactivity was detected with an enhanced chemiluminescence kit.

### Image analysis

2-DE image analysis was performed using PD-Quest software (Bio-Rad) according to the manufacturer's instructions. The normalization of each individual spot was performed according to the total quantity of valid spots in each gel, after subtracting background values. The spot volume was used as the analysis parameter to quantify protein expression.

### In gel Digestion and MALDI- TOF analysis

Coomassie G-stained spots were excised from 2-DE gels; destaining and in-gel enzymatic digestion were performed as previously described [28]. Briefly, each spot was destained with 100 µl of 50% v/v acetonitrile in 5 mM ammonium bicarbonate and dried with 100 µl of acetonitrile. Each dried gel piece was rehydrated for 40 minutes at 4°C in 10 µl of a digestion buffer containing 5 mM ammonium bicarbonate, and 10 ng/µl of trypsin. Digestion was allowed to proceed overnight at 37°C and peptide mixtures were stored at 4°C until assayed. All digests were analyzed by MALDI-TOF (TofSpec SE, MicroMass) equipped with a delayed extraction unit. Peptide solutions were prepared with equal volumes of saturated α-cyano-4-hydroxycinnamic acid solution in 40% v/v acetonitrile-0.1% v/v trifluoroacetic acid. The MALDI-TOF was calibrated with a mix of PEG (PEG 1000, 2000 and 3000 with the ratio 1∶1∶2) and mass spectra were acquired in the positive-ion mode. Peak lists were generated with ProteinLynx Data Preparation (ProteinLynx Global Server 2.2.5) using the following parameters: external calibration with lock mass using mass 2465.1989 Da of ACTH, background subtract type adaptive combining all scans, performing deisotoping with a threshold of 1%. The twenty-five most intense masses were used for database searches against the SWISSPROT database (release 2011-01 of 11-Jan-11) using the free search program MASCOT 2.3.02 (http://www.matrixscience.com). The following parameters were used in the searches: taxa Homo sapiens, trypsin digest, one missed cleavage by trypsin, carbamidomethylation of cysteine as fixed modification, methionine oxidation as variable modification and maximum error allowed 100 ppm. Only proteins with a Mascot score >55 were taken into consideration.

### Statistical analysis

Data from image analysis were used as values of protein expression in 2-DE experiments as previously described [29]. The two-tailed Student's t test was used to verify the significance of the variations of expression of proteins in HI-MSCs and BM-MSCs cells, control and differentiated cells. Experiments were performed in triplicate. Statistical significance was set at p-values≤0.05 (Tables 1, 2, 3). In 2-DE experiments proteins were classified as differentially expressed if the ratio of the spot intensity between treated cells and control cells was greater than 1.5-fold (protein overexpressed) or lower than 0.5-fold (protein underexpressed). For proteins expressed by all cell populations the ratio is calculated between value of the basal cells (HI-MSCs, BM-MSCs) compared to the mean value of differentiated cells (HI-ILCs, BM-ILCs).

### Hierarchical Cluster Analysis

The expression profile of the identified proteins was analyzed with hierarchical cluster analysis to underline similarities between proteins as previously described [30]. The analysis was performed with R software 2.11.1 [31] and the package gplots 2.8.0 [32]. The distance matrix was obtained with the Euclidean method, and the clustering with the furthest neighbour method. The heat map was obtained with the default options contained in the package function and adapted to mark expressed and unexpressed proteins with black and white.
